# A Qualitative Investigation of the Impact of COVID-19 on United States’ Frontline Health Care Workers and the Perceived Impact on Their Family Members

**DOI:** 10.3390/ijerph191710483

**Published:** 2022-08-23

**Authors:** Gary Edward Schaffer, Lisa Kilanowski, Brian En Chyi Lee

**Affiliations:** 1 Department of Counseling and School Psychology, College of Education, Niagara University, Lewiston, NY 14109, USA; 2 School of Psychology, Deakin University, Melbourne, VIC 3125, Australia

**Keywords:** frontline, health care worker, family functioning, family dynamic, COVID-19, pandemic

## Abstract

Although previous research has documented the mental and physical health impacts that COVID-19 had on frontline health workers in the United States, little is known about how the pandemic affected their families. This study sought to explore the impact COVID-19 had on the individual functioning of frontline health care workers in the USA and the perceived impact it had on their family members during the initial nine months of the pandemic. More specifically, this study sought to explore if and how family roles, routines, rules, and social-emotional well-being changed as a result of COVID-19. Twenty-eight frontline health care workers across the United States who were parents to at least one child residing in the home under 24 were interviewed. Data were analyzed using reflexive thematic analysis. From the analysis, four major themes emerged with regard to the changes and perceived impact to family functioning, family experiences of new hygiene practices, and stigma related to being a health care practitioner or having a family member working in health care, and psychological distress. The results of this study can be used by mental health clinicians to inform policy, develop practice guidelines, and help identify and target interventions for health care workers and their family members.

## 1. Introduction

The global spread of the coronavirus disease (COVID-19) in December 2019 and into 2020 led to unprecedented hardships for the nearly 22 million health care workers in the United States [1,2]. Despite the best efforts of these professionals to successfully treat and provide comfort for children and adults with coronavirus, the disease continued to proliferate. Just eight months after the World Health Organization (WHO) declared COVID-19 as a pandemic on 11 March 2020, there remained over 12,000,000 cases and 255,000 deaths from COVID-19 in the United States alone [3,4]. Not surprisingly, due to the overwhelming strain COVID-19 placed on frontline health care professionals, many reported fears and frustrations throughout the pandemic with regard to poor organizational management, lack of personal protective equipment, being overworked, facing high emotional demands, contracting the disease, and being stigmatized and betrayed by members of the public not employed in health care [2,5,6,7,8,9,10]. Consequently, an emerging literature base has found that frontline health care workers during COVID-19 reported feelings of increased stress, anxiety, loneliness, isolation, depression, PTSD, and sleep disturbance [2,5,6]

Although a literature base has emerged on the occupational and emotional strain COVID-19 has had on health care workers, little research has focused on how the pandemic impacted family relationships and cohesiveness for these professionals. While the pandemic and social distancing measures may have provided opportunities for families to improve their relationships, it may have also caused increased conflict and discord due to members spending more time together or having to follow more rigid rules and routines such as increased hand washing [11]. For the purposes of this study, social distancing and public health measures can be defined as a series of protective strategies put into place by government or businesses that restrict the movement and gathering of most community members in an effort to reduce the spread of COVID-19 [8,11]. Examples of social distancing and public health measures used during COVID-19 include having people maintain at least six feet of distance from others, advising that people avoid crowds or large gatherings, temporarily closing schools and businesses, and requesting that people work from home instead of heading into the office [12]. Unlike other professions that could work from home during social distancing to protect themselves from contracting COVID-19, frontline health care professionals were required to be present at work and were consequently at higher risk of contracting the disease themselves or spreading it to family or community members [8,13]. Outside their professional roles, frontline health care workers still have their familial duties of being a parent and possibly a spouse or significant other.

### 1.1. Existing Studies on Frontline Health Care Workers and COVID-19

At present, few studies have evaluated the impact of COVID-19 on the families of frontline health care workers. Through the studies that exist, several themes have emerged with regard to increased family time together and cohesiveness, loss of personal space and privacy, hypervigilance and worry of infecting others, increased levels of parent–child conflict, and alterations in family roles and routines [8,14]. For example, in a qualitative analysis of 39 frontline health care workers from Victoria, Australia, Sheen et al. (2022) found that some participants were challenged by the need to hand over existing roles and responsibilities to other members of the family due to their increased demands at work during the pandemic. Additionally, frontline health care workers expressed difficulty in balancing work and parenting and expressed concern over their children’s mental health and well-being. 

On another note, health care workers relayed concerns over bringing COVID-19 home to their significant other or children, which led to feelings of isolation and a loss of social connection with friends and extended family members. These qualitative findings were similar to those found by Amakiri et al. (2020) in which in the United Kingdom, families of health care workers reported feelings of uncertainty and loneliness [15]. Due to familial relationships often serving as either pillars of strength and/or a taxing relationship during times of stress, it is important to further understand if and how the COVID-19 pandemic impacted family roles and functioning for frontline health care workers [10]. In the context of this study, family functioning includes an understanding of family, routines, roles, and rules, along with the challenges and strengths.

Previous research suggests that family functioning has the power to help and/or hinder an individual’s ability to cope with stressors during major life events such as COVID-19 [16,17,18]. Resilient families have the ability to withstand and rebound from disruptive life changes, while less resilient families may experience increased stress and turmoil [17]. Moreover, stressors that impede the functioning of one family member may lead to alterations in functioning for all family members [19]. Therefore, if a stressful life event such as COVID-19 is greatly impacting the functioning of one individual in the family, it may have an effect on other family members.

One theory that suggests that serious life events and challenges such as the COVID-19 pandemic impact the whole family is that of family systems theory [20]. In brief, family systems theory suggests a family is a collection of people who are integrated and are interdependent on each other [21]. Consequently, external environmental disruptions such as COVID-19 can lead to alterations in family functioning across family sub-systems such as the co-parenting unit, family–child unit, and the large extended family unit [21]. Previous research has highlighted the impact of COVID-19 and other environmental stressors such as parental conflict has had on family functioning [17]. In particular, studies on COVID-19 and family functioning have highlighted that while the pandemic may have increased feelings of connectiveness and communication among family members, there was also increased feelings of tension and conflict with regard to hygiene practices, social distancing and quarantine protocols, and employment alterations [17].

To date, no studies have explored how COVID-19 has impacted the individual functioning of frontline health care workers in the USA and how they perceived the pandemic had impacted their family members during this period of time. However, it is important to investigate how the pandemic impacted both frontline health care workers in the USA and the perceived impact it had on their family members to best provide support and services. Understanding individual and family functioning during stressful events has the power to help or hinder an individual’s attempt to process and cope through stressful life events such as the COVID-19 pandemic and can be used by clinicians to identify and target interventions [16]. The results from this study can also be used to inform policy, professional development, and practice guidelines for this critical component of the workforce

### 1.2. Aims

The current qualitative study sought to evaluate how the COVID-19 pandemic impacted the individual and perceived family functioning of frontline health care workers in the USA. More specifically, the current study sought to explore the views of frontline health care workers on if and how family roles, routines, rules, challenges, strengths, and social-emotional well-being changed within the initial nine months of the pandemic. The research questions for this study were:

(1) According to health care workers, what was the experience of their families during the pandemic and associated measures?
(2) Did the family roles and responsibilities of frontline health care workers change during the pandemic? If so, how? If not, why not?
(3) Did health care workers or their family members experience any psychological distress due to COVID-19?

## 2. Materials and Methods

### 2.1. Data Collection Materials

Ethical approval for this study was provided by the Niagara University Institutional Review Board (IRB #2020-046). Participants completed one semi-structured interview, which ranged from 32 to 126 min, with an average interview taking 64 min. The initial semi-structured interview schedule was developed by Sheen et al. (2022), and with approval by the authors was utilized and modified to best elicit responses from frontline workers in the USA [8]. For example, a modification made from the initial survey included removing the word “lockdowns” from questions (a measure employed in Australia) and substituting the term “social distancing measures” (more likely to be used in the USA). For the purposes of this study, social distancing and public health measures were defined as a series of protective strategies implemented by governments or businesses that restrict the movement and gathering of community members in an effort to reduce the spread of COVID-19 [3].

Examples of questions asked in the interviews included “has anything changed for your family since social distancing measures were put into place?” and “how, if at all, did your family support each other when social distancing measures were put in place? Was this support different from pre-COVID times?” Interviews with participants were completed between September 2020 and December 2020. All interviews were completed via telephone, then transcribed. Participants were sent the transcript with an invitation to remove potentially identifiable information, correct any of the information presented, or add anything they considered important. Participants were given two weeks to provide feedback on their transcript with no changes made. 

### 2.2. Participants

The study participants were 28 frontline health care workers across the USA employed in hospitals only treating COVID-19 patients as well as COVID-19 hospital wards, intensive care units, emergency departments, and physician’s offices. Twenty-four of the participants were female and four were male, with at least one child living with them under 25 years of age at the time of the study. Participants ranged in age from 30 to 55 years with the mean age being 39.3 years. Participants had one to four children residing in the home with them (M = 3.64) ranging from 3.5 months to 23 years of age. All participants were at risk of being exposed to suspected or confirmed COVID-19 cases while performing their professional duties at work.

See Table 1 for further demographic details.

### 2.3. Procedure

Recruitment advertisements for this study were posted to online social media platforms such as Facebook and LinkedIn and on health care professional and parenting group pages such as COVID-19 Nurses, Physician Assistants, Respiratory Therapy Breakroom, The Break Room Support for Healthcare Professionals, Gentle Parenting, and Parenting Support Group. The email address of the principal researcher was provided in both advertisements and on social media. Interested participants emailed the principal researcher and interviews were then coordinated. In total, 28 interested participants across the United States emailed the principal researcher and completed the interview for the study. Participants who took part in this study must have been frontline health care workers who were actively working with patients in health care settings in which they could be exposed to COVID-19 (i.e., hospital, nursing home, doctor’s office, etc.).

In an attempt to recruit culturally diverse health care professionals, advertisements were posted to diversity, inclusion, and justice social media pages and to pages supporting culturally diverse health care practitioners. Upon the completion of each interview, each participant was encouraged to inform other health care workers to email the author. Follow-up emails were sent to health care practitioners who took part in the study. These emails contained information and a pdf flyer with information on the study that they could forward onto their co-workers in health care.

### 2.4. Data Analysis Procedure

A reflexive thematic analysis was used to analyze the data. Reflexive thematic analysis is an approach employed to elicit people’s experiences, representations, and views and perceptions regarding a given phenomenon [22,23]. Within this approach, themes are conceptualized as meaning-based patterns evident in both conceptual and explicit ways (Sheen et al., 2022). Reflexive thematic analysis allows the researcher to determine the focus of the work as opposed to being bound to a specific theoretical framework, which can reduce the loss of the individual participants’ experiences and perspectives during an unusual event such as COVID-19 [22]. Reflexive thematic analysis was completed in an idiographic manner by analyzing each interview transcript before comparing emerging themes across the sample. 

The first author preliminarily analyzed each transcript, coded the participant responses, and identified quotes that were meaningful to the research questions on a micro (individual transcript) level. A second author then reviewed these themes, along with a close read of each transcript, and together, they compared and contrasted themes across transcripts. The transcripts were then reviewed by all, with consensus being reached on themes through discussion and the rechecking of transcripts. The participants’ gender, age of children, occupational role, geographic, and other demographics were considered in the development of themes and reported when relevant in the results. Through completion of reflexive thematic analysis, four major themes emerged with regard to changes and impact to family functioning, family experiences of new hygiene practices, stigma related to being a health care practitioner or having a family member working in health care, and psychological distress. Overall, this study utilized a phenomenological methodological orientation in which there was a focus on how participants in this study made sense of their lived experiences throughout the COVID-19 pandemic and how it impacted their family.

## 3. Results

Using reflexive thematic analysis, we identified four superordinate themes and nine subordinate themes. The thematic structure is displayed in Table 2. 

### 3.1. Changes and Impact to Family Functioning 

In the context of this study, a major theme emerged with regard to the changes and frontline health care worker’s perceived impact to family functioning. Specifically, the participants clearly expressed changes with their family cohesion and unity. However, while changes were evident, there were diverging directions of change in family cohesion and unity, with some families experiencing changes that helped them grow closer, but others experiencing growing conflict. 

#### 3.1.1. Growing Closer

The pandemic was typically identified as a time where many families grew closer and/or experienced increased tension. When comparing family support before the pandemic to family support during the pandemic, some participants described improved family cohesion, increased communication, and a greater willingness for family members to help one another out. To illustrate, one frontline nurse remarked:

*I think this ordeal has brought my boyfriend and I closer. I was really able to lean on my boyfriend as a couple. My boyfriend knew what I needed and started taking care of the stuff that needed to get done around the house before I got home.*

Additionally, some participants described how their children grew closer to each other, with one nurse noting, *“Watching my kids become closer is one of the blessings of COVID”.* Not only did the participants remark on how their children grew closer as siblings, but they also noted that they became more independent with one nurse stating, *“We now rely on the 11-year-old to get breakfast and complete chores. My daughter now makes pancakes for the whole family or bakes stuff for the whole family”.*

Some participants remarked that one of the reasons their families grew closer together was because they spent more time together as a result of not filling their schedules with activities. One nurse remarked, “*There is a team mentality that we are in this together. We are more together as a family. I think that it is because we are not overbooking our schedules now*”. Other participants noted how their families filled their schedules with more family-oriented activities such as going on walks, watching movies together, or eating dinner together. One nurse indicated, *“Since COVID began, we have all taken an interest and watch things together on television together. My son and I have now gotten my wife to come out mountain biking with us”.*

Some participants remarked on having more open and honest conversations with their partners with one nurse stating, “*I told my wife and showed her how to pay the bills in case I got sick with COVID-19. The more I think about it, the more I think we should have had these conversations before because my wife will need to know how to pay the bills whether it be from me getting sick with COVID or getting into a car accident*”.

#### 3.1.2. Growing Conflicts

Although many families reported growing closer, some participants also reported increased tensions resulting from financial concerns due to the pandemic, unilateral parenting decisions, or lack of privacy. The quotes below highlight some of the tensions experienced by families:

*My husband had to shut down his business during COVID-19, and he was extremely worried because he had a lot of overheads. It took a major hit on my husband’s mental health. There was definitely tension in the house because I wanted to be at home, and my husband wanted to be at work (COVID-19 Nurse Manager).*

*My husband pulled my son right out of preschool during COVID. I personally felt a little frustrated that my husband wanted to do it so quick (Respiratory Therapist).*

*I guess I am more restrictive with the kids and what they can do. I would rather have them not do some things and go some places. My husband is less weary than I am about it so that can create some tension (Nurse).*

*I think there is some tension in the marriage because the kids are around more. My husband and I are not getting as much alone time together and individually because the kids are around. (Nurse).*

*During COVID-19, my daughter really lived and stayed upstairs and I stayed downstairs. My daughter would become upset if I came upstairs, and she got a lot more privacy than she usually did (X-Ray Technician).*

### 3.2. Individual and Family Experiences of New Hygiene Practices

Throughout the interview, participants expressed how their families adopted new hygiene practices due to the pandemic and as a result of them working in health care. Overall, the participants described a sense of hypervigilance in their social interactions with their family and extended family. 

#### 3.2.1. Isolating for Family Safety

Participants described many instances where they had to self-impose isolation and social distancing practices to keep their immediate family safe. Some participants noted that they reduced or significantly limited their children’s physical interactions such as hugging family members. For example, one physician assistant stated, “*You can see it in their grandparents eyes when they wanted to give my children hugs, and we had to keep it to fist bumps. It was difficult trying to explain not hugging your grandparents to a three, four, and one-year-old.*”. Similarly, another nurse manager stated, *“Since COVID-19, I now tell my children’ you cannot eat out of my popcorn bowl.’ I also tell them that ‘you have to sit on the ground and not on the couch or on the floor with me.’ At night, my son would ask why I could not hug and kiss him.”.*

Other frontline health care workers highlighted how they stayed in a hotel to avoid exposing their family to COVID-19. One nurse noted, *“I would quarantine for ten days in a hotel and then I would go back to my kids and husband for three weeks. After being home for three weeks, I would start the process again.”.* Another respiratory therapist would not allow her family to travel in her car stating, “*My car has become the COVID car. I no longer take my car with the family in it that much anymore due to COVID. We take my husband’s car*”.

To protect their family from possible exposure to COVID-19, some participants also noted that they began to isolate themselves from extended family members who were not following proper social distancing and public health protocols. For example, one nurse revealed:

*My parents went to a casino and I was like are you kidding me? My mom said we are just having fun, so I said you cannot see your granddaughter for 14 days. She was like ‘why would you take her away?’ I said I don’t like the decisions you have made.*

Other participants reported that they isolated themselves and their children from extended family members due to fear of having an at-risk family member contract COVID-19. One nurse indicated, “*The kids used to go to their grandfather’s every Saturday. But they did not see their grandfather from April until school started up again because their aunt who resides with their grandfather and has severe asthma.*”. Finally, some participants noted disagreements with their parents over hygiene practices and the treatment of COVID-19. One physician noted, *“My parents will tell me hydroxychloroquine works to cure COVID, and I will tell them do not get your COVID-19 information from the news”.*

#### 3.2.2. Breaking the Bridge between the Home and the Workplace

Bringing home the COVID-19 virus and conversely, bringing COVID-19 to work, was a strong concern for many participants, leading them to adopt increased hygiene practices and hypervigilance. Specifically, some participants adopted a routine of either changing immediately out of their clothes upon coming home from work and washing them or bringing another pair of clothes to work. One nurse remarked, “*I wore regular street clothes into the hospital, and then I would change into the scrubs at the hospital. Then I would leave my scrubs at the hospital and get re-changed into street clothes.*”. Other participants remarked on ruminating thoughts of passing COVID-19 onto their family members with one director of clinical research and respiratory therapist stating, *“You think about how it can affect the patient, you, or your family. You don’t want to bring it home to your family.*”. To avoid exposing patients to COVID-19, one physician’s assistant remarked on how their husband *“ran out to do errands so that I would not catch COVID from the public and so that I would not unknowingly expose patients”.*

#### 3.2.3. Mitigating Risks of Family Infections

The participants and their families were also worried and concerned about the risk of infection within the home and took precautionary measures to mitigate this risk. When communicating with family members in their household, some participants highlighted how technology was utilized to limit the potential exposure to COVID-19 and adapting their parenting practices to allow this to happen. For example, one nurse stated, “*The girls text and facetime me more from their rooms in the house, which I used to never let them do.*”. Moreover, other participants remarked on how they taught their kids better hygiene practices with one nurse manager stating, “*I taught my kids to buy spray hand sanitizer because it is usually higher in alcohol content. We are washing our hands and using sanitizer more as a family.*”. It was not only the participants who were concerned about infecting their family, but their partners also acted on these concerns; a respiratory therapist noted that their *“husband goes around when I get home and wipes down and bleaches everything that I touch, such as doorknobs, countertops, the shower knob, and the car.*”. Finally, one physician noted purchasing a product to minimize COVID-19 exposure stating, *“So my father-in-law has part ownership for a product that creates a sterile environment that plugs into the ductwork. We had that installed in our house, and we were pretty happy with it*”.

### 3.3. Stigma and Alienation

Within this theme, the participants noted their own and their family members’ experiences with stigma and alienation from the general public, family, or neighbors due to their work as a health care worker. 

#### 3.3.1. Stigma as a Health Care Worker

Some health care professionals remarked on how they could not go out in public to provide for themselves with one nurse noting, *“I went to the supermarket on the way to work with my scrubs on to pick up something and some gentleman told me to ‘get the hell out of the store’ because I was bringing COVID in.”*. Another nurse remarked on how it was difficult for her to find a *“hairdresser who would work with me because I am a nurse. My former hairdressers that I would use would not do my hair*”.

While some health care professional experienced difficulty providing for themselves or obtaining basic cosmetic services such as a haircut, others remarked on how they no longer maintained communication with extended family members due to COVID-19. One nurse stated:

*My extended family is very judgmental. My brother and my brother and sister-in-law have accused me of lying about COVID. I no longer talk to my brother-in-law because he got into it with me over COVID-19 at a family gathering. He thought I was profiting off of COVID-19 and the health care industry was profiting off of COVID-19. I am no longer allowing my kids around my brother-in-law and his kids because of COVID and my brother-in-law’s views towards COVID.*

#### 3.3.2. Stigma as a Family Member of a Health Care Worker

Even when some health care professionals did not report disruptions in their personal relationships with extended family members, they remarked on how working in health care indirectly impacted others they were related to, with one nurse stating, *“when my sister’s work found out that I was a COVID nurse they asked my sister to stay home from work.”.* Out of fear for how the general public would react, some participants indicated that they told their children not to inform others that they worked in health care. One nurse manager noted, *“I stopped telling people that I was a nurse in public. I told my kids to stop telling people that I was a nurse because people were afraid of me because of potential exposure to COVID-19.”.* Other participants remarked on how their children were stigmatized due to their parent(s) working in health care. A respiratory therapist remarked, *“My daughter has a best friend that lives across the street and they are both teachers, and she told my daughter that I am not allowed to play with you because your parents work with COVID patients”.*

### 3.4. Psychological Distress

During the initial months of the pandemic, frontline health care workers in this study reported experiencing increased stress, anxiety, loneliness, isolation, sadness, rumination of negative thoughts, trauma, and sleep disturbance. One nurse remarked, *“My mental health has significantly declined since COVID-19. I don’t feel as organized or together.”.* Many of these feelings they attributed to feeling helpless in saving people’s lives from COVID-19 or not understanding why one person survived and another passed away from the disease. One nurse highlighted the struggle of watching one person survive COVID-19 and another pass away saying:

*So there was a gentleman the same age as my husband and had two kids. We were going to have to intubate him and hook him up to a ventilator. He worked as a nurse’s aide. He said to his wife “everybody is intubated. I hope I make it through.” There was a lot of uncertainty if he would survive. He survived. Another gentleman said “can you move my grandkids pictures in front of me so I can see them when I wake up.” He never woke up and passed away.*

#### 3.4.1. Sources of Psychological Distress

Workplace safety was described as a factor related to stress, with a nurse noting how not having proper personal protective equipment caused them added stress while at work saying, “*during the peak of COVID, we did not know that much about the disease and what was going to happen and there was not enough PPE for everyone and it was kinda stressful because the policies kept changing. You didn’t feel safe at the hospital*”.

Other frontline health care professionals remarked on feelings related to moral distress. One participant described wanting to do better for the patients with one respiratory therapist stating that there were “*some horror stories and lonely nights in which you think to yourself whether I could have done things better for the patient. There were times I was crying in the shower for the patients and whether I could have done better*”. Other health care professionals reported watching people they knew pass away, with the same respiratory therapist revealing, *“My friend’s niece actually ended up in the hospital at age 22. I took care of her and she made it 47 days and eventually died*”.

Surprisingly, some participants in this study remarked on how their own family negatively impacted their social-emotional well-being with one nurse saying:

*It is very frustrating when people think COVID is a hoax, and my own father said it was a hoax. I told my father that I work in a hospital and I am watching people dying, and these folks are watching television saying it is a cold. I told my wife that I may never talk to my father again. It has added more strain to my family relationships.*

Against these mental health issues, some participants indicated how they were coping with COVID-19 with one nurse saying, “*I have went on anti-depressants and doubled the dose on my sleep medications. I am now prescribed anxiety medication as well (Xanax). I take it as needed. I think I now have PTSD because of COVID*.”.

#### 3.4.2. Concerns for Children’s Mental Health

Finally, some frontline health care workers remarked on the psychological distress the pandemic had caused their children and expressed concern over the lack of available mental health care for youth. One nurse stated:

*I think that there will be a one-thousand percent need for more children’s mental health clinicians in New York State. To get my daughter into a counselor during COVID took two weeks, but then my daughter was referred to a counseling service for additional services that took up to three weeks. Within the five weeks that she was waiting for services, my daughter was hospitalized and seen in the emergency psychiatric unit at the hospital. I think that might have been prevented if my daughter had access to children’s mental health services right away.*

Other participants reflected on how the lack of socialization may be impacting their children or family’s mental health as a whole. With regard to her daughter, one nurse remarked, *“I think it is going to affect her because she is lacking socialization and is only seeing her peers and teacher over the computer.”* Another respiratory therapist noted, “*We used to be more social. Now we do everything at home. Our entertainment is television and video games as opposed to going out*”.

## 4. Discussion

### 4.1. Family Cohesion and Closeness

The present qualitative study explored the impact that COVID-19 had on health care workers and how they perceived it impacted their family dynamic and family members during the initial nine months of the pandemic. The participants in this study reported common themes as a result of the pandemic affecting intrafamilial functioning and psychological distress. The first theme that emerged was increased family cohesion and closeness, both in isolation and in conjunction with increased family tension. Implications of this theme extend beyond the pandemic. Due to COVID-19 restrictions limiting engagement in activities outside of the home, it appears that families were forced to abandon the potentially deleterious culture of the frenetic overscheduling of activities common in American households [18]. This appeared to increase the opportunities for familial bonding and engagement. Therefore, the COVID related implications of this finding may serve to buttress extant and conflicting research on the impact of overscheduled children and families [24,25].

The results of this study also revealed that there was a reported increase in reliance on same-household family members to complete chores and run errands that they either did not complete before the pandemic or did so to a lesser degree. Reliance on same-household members to complete chores or errands stemmed from social isolation as a result of working in health care, working increased hours during the pandemic, or due to the stigma and alienation many health care workers experienced when engaging with the general public such as when shopping for groceries. These findings were similar to those by Sheen et al. (2022), who reported that family members of health care workers assumed additional responsibilities during the initial months of the pandemic [8]. 

The identification of the degree to which such increases in cohesion and connectedness served as a protective factor against other COVID-19 related stressors was not ascertained in the current work, but is plausible given extant research [26,27]. In many cases, increased family tension appeared to coexist alongside increased cohesion, spurred by an array of challenges ranging from financial difficulties to differences in parenting decisions and an overabundance of time together without the option for socialization outside of the home. Future work in this domain should seek to identify the unique contribution of external stressors such as financial challenges and the lack of socialization outside of the family unit, on family reports of increased cohesion, increased tension, or a combination thereof. The use of valid and reliable measures related to stress during the pandemic [2] may prove beneficial in tandem with qualitative approaches. 

### 4.2. Hygiene Practices and Family Relationships

Adherence to strict hygiene practices intended to limit the spread of COVID-19 within and outside of the home were repeatedly linked to disruptions in routine family functioning among first degree and extended family. Within the home, physical distancing of health care workers from children and partners, lack of physical touch and proximity, and the adoption of new bonding rituals and routines to maintain familial intimacy in the absence of proximity or physical touch were universally reported. The cited disruptions among health care workers appeared to exist along a continuum, with distancing-oriented hygiene practices allowing them to stay in the home (e.g., changing immediately upon return from work, spending time physically apart within the same residence, using alternate vehicles, texting each other from different rooms, fist bumps instead of hugs) presenting as less disruptive than those entailing the time spent in hotels or alternate locations for extended periods of time. The self-created alternate bonding methods employed by families during distancing illuminate the inherent resilience of some family units, while also shedding light on the utility of providing targeted bonding and intimacy maintenance strategies to struggling families via coaching, counseling, and psychoeducational resources [27]. 

Hygiene practices also led to disruptions to the health care workers’ relationships with extended family, with the unique identification of themes related to differences of opinion on COVID-19 transmissibility, severity, and lack of need for health safety practices among extended family emerging as a source of divisiveness and discord. Overt conflict over the topic of whether COVID-19 was “real” or “a financial ploy”, precautions, and transmissibility was cited by several participants as having long-term and negative implications for extended family interactions. It appears that this conspiracy-oriented disbelief in COVID-19 was juxtaposed with the separate theme of stigma and alienation from extended family and others as a result of employment as a health care worker in regular contact with the virus. Conspiracy-oriented disbelief was not apparent and not reported as a theme in the study by Sheen et al. (2022) [8]. 

### 4.3. Psychological Distress 

Psychological distress, inclusive of both frontline health workers and their children, emerged as one of the most significant themes in the relationship to public health needs and actionable public health imperatives. Multiple thematic layers related to acute psychological distress and traumatic responses (e.g., inability to relax at home, ruminating thoughts of work, reduced sleep, nightmares) were revealed across participants, indicating a need for proactive and targeted protocols for providing mental health support to frontline health workers. Such findings are consistent with those of Sheen and colleagues (2022) [8], suggesting that psychological distress directly linked to employment as a frontline health care worker is not exclusive to health care work in the United States or a function of the structure of the American health care system. Though multiple avenues for pursuing mental health support were made available to health care workers in the United States later in the progression of the pandemic (e.g., Heroes Health Initiative, National Alliance on Mental Health Frontline Professionals Resources, The Well-Being Initiative, among others), the findings from this study suggest that immediate, cohesive, and planful linkage to such resources by employers is imperative during health care crises. Though a robust number of online, informational resources are available for frontline health care workers, coordinated access to active interventions such as online or work site counseling and support groups may best mitigate the cumulative and progressive effects of psychological distress during periods of crisis amidst uncertainty. 

Thematic layers reflective of health care workers concerns for the mental health and well-being of their children as well as overt reports of children presenting with acute and sub-threshold mental health needs may suggest a bi-directional influence of pandemic related workplace trauma on both the caretakers and children. Though further research is necessary to identify the relationship between parental mental health during the pandemic and the psychological functioning of children, reports of mental health concerns among children explicitly linked to the pandemic in isolation, combined with notably excessive wait-times for mental health support, necessitate the future development of protocols to provide such support to children in distress during times of global crisis. Given the chronic deficit of accessible mental health services for children predating COVID-19 [28,29,30], the current findings are of even greater significance, and underscore the need to increase the accessibility to mental health services in arenas currently serving children such as schools [31]. 

## 5. Limitations

The current study was largely comprised of Caucasian participants working in the Northeastern United States, with smaller numbers of participants from the Southeastern, Southwestern, and Midwestern regions. This may be due to the pandemic hitting this region of the country first and hardest, with more than two-thirds of COVID-19 deaths in the United States occurring in the Northeastern states during the initial months of the pandemic [32]. Another limitation of this study involves the unevenness of the age distribution of the participants, with the greatest number of participants resting between 30 and 39 years of age. This uneven age distribution leaves the experiences of those in both the early career and late career stages somewhat unaccounted for. 

As is common among health care workers in the USA, Caucasian females comprised the majority of respondents [33], with the greatest number of participants identifying as nurses and respiratory therapists. Though not representative of the full range of frontline medical professionals, the slight overrepresentation of respiratory therapists provides valuable insight into the experiences of the families of health care workers assisting the most critical patients during the COVID-19 pandemic. Likewise, the skew toward married participants with children, though unintentional, served in our efforts to elicit feedback inclusive of family functioning and family relationships. Though an interview of the respondent partners and children was sought, the number of family members consenting to family interviews was too limited (significant others and spouses *n* = 4; children *n* = 7) for inclusion. Consequently, data garnered from this investigation represent the experiences of the primary respondent and their perceptions of the family experiences. To more fully document the impact of COVID-19 on family functioning in the future, a direct interview of the partners of health care workers and their children is recommended.

Given the principal findings of this inquiry, future investigations of the experiences of frontline health care workers and family relationships should feature more targeted qualitative and quantitative approaches. Direct assessment of family functioning and mental health using valid, reliable, norm-referenced assessments, combined with a precise line of qualitative questioning targeting relational issues and mental health, may yield more robust insight that can be normatively qualified. The inclusion of questions that involve extended family and elder care responsibilities is also recommended to more fully capture the scope of family relationships, obligations, potential stressors, and sources of support.

## 6. Implications and Conclusions

The findings of the present work are consistent with the central tenets of family systems theory [20,34], which suggest that external environmental disruptions such as a global pandemic or another substantial environmental disturbance (unemployment, changes in work status, illness) can lead to changes in family functioning and balance across family sub-systems, inclusive of the co-parent unit, family–child unit, and the larger extended family unit. In the case of the current exploratory study, environmental disruptions in the form of COVID-19 related hygiene practices, social distancing and quarantine protocols, employment alterations (e.g., working long hours, working in close contact with COVID-19 patients), and COVID-19 fear and disbelief were indeed found to impact global family functioning across units. However, in many cases, the cited disruptions were noted alongside of or in contrast to the reported increases in familial bonding, leading to evidence of both distress and increased cohesion within some family systems. Additional quantitative research identifying the unique contextual, intra/inter-individual, and psychological (e.g., psychological flexibility, inflexibility, and combinations of both) [21] factors within each family unit is needed to further understand the complexities of family functioning during times of a global health crisis, in accordance with family systems theory. 

Implications of the present work support the need for additional research on the impact of COVID-19 on extended family relationships alongside the need for increased access to targeted, direct-service mental health intervention for children and families in times of a global health crisis. Previously undocumented findings related to the impact of COVID disbelief on family relationships bear implications for the adherence to public health mandates such as increased hygiene and social distancing as well as the maintenance of familial bonds during times of pandemic-related uncertainty. Though international research has not extensively focused on the prevalence of COVID disbelief and skepticism, a high proportion of Americans continue to espouse attitudes consistent with COVID disbelief and disinformation [35,36]. Exploration of the short- and long-term impact of health care workers’ interactions with “skeptical” family and the community at large using validated tools such as the COVID-19 Disbelief Scale [37] may yield insights into the breadth and depth of familial disruptions stemming from the disbelief and lack of responsiveness to public health imperatives. 

While the mental health needs of front line health care workers have been referenced in other pandemic related inquiries (e.g., Sheen et al., 2022 [8]), parental concern regarding the mental health needs of children of health care workers during the pandemic emerged as an equally strong theme in this current work. Given the documented shortage of mental health providers serving youth in the United States predating the pandemic [28,29,30], combined with documented increases in mental health needs as rooted in the pandemic [38], bolstering accessible mental health support for children is imperative. Increasing access to remote school-based mental health services, provided by school psychologists, school counselors, and social workers, may serve as the gateway for immediate and consistent mental health services for the youth. Additionally, state legislators can greatly expand access to children’s mental health services by allowing for the licensure of specialist-level school psychologists so that they can practice outside the four walls of the schools [39]. Overall, greater support is needed and should be offered for frontline health care workers and their family members as they continue to cope with the impacts of COVID-19 on their personal and professional lives.

## Figures and Tables

**Table 1:** The participants’ demographic information (*n* = 28).

	Frequency (*n*)	(%)
Gender		
Female	24	85.7
Male	4	14.3
Ethnicity		
Caucasian	26	92.9
Indian	1	3.6
Mixed	1	3.6
Age range		
30–39	18	64.3
40–49	9	32.1
50–59	1	3.6
Geographic location in U.S.		
Northeast	17	60.7
Southeast	5	17.9
Midwest	4	14.3
Southwest	2	7.1
Level of education		
Associate’s degree	8	28.6
Bachelor’s degree	14	50.0
Master’s degree	5	17.9
Doctoral degree	1	3.5
Occupation		
Nurse	11	39.3
Respiratory therapist	9	32.1
Nurse manager	3	10.7
Nurse practitioner	2	7.1
Medical doctor	1	3.6
Physician’s assistant	1	3.6
X-ray technician	1	3.6
Relationship status		
Married	20	71.4
Divorced	5	17.9
Single	2	7.1
Divorced	1	3.6
Age range of children		
1<	1	2.1
1–2 years	4	8.3
3–6 years	13	27.1
7–10 years	11	22.9
11–14 years	14	29.2
15–18 years	4	8.3
19–21 years	0	0
22–25 years	1	2.1

**Table 2:** Identified themes and subthemes.

Changes to Family Functioning	Growing closer Growing conflicts
Individual and Family Experiences of New Hygiene Practices	Isolating for family safety Breaking the bridge between the home and the workplace Mitigating risks of family infections
Stigma and alienation	Stigma as a health care worker Stigma as a family member of a health care worker
Psychological Distress	Sources of psychological distress Concerns for children’s mental health

## Data Availability

The raw, de-identified data supporting the conclusions of this paper will be made available by the authors without reservation.
